# Atrioesophageal fistula with meningitis: A rare complication of atrial fibrillation ablation, case report and literature review

**DOI:** 10.1016/j.amsu.2020.07.019

**Published:** 2020-07-16

**Authors:** Kayle Warren, Pratishtha Singh, Austen Stoelting, Shawn Esperti, Kunjan Udani, Thaddeus Golden

**Affiliations:** Department of Internal Medicine. Grand Strand Medical Center, Myrtle Beach, SC, USA

**Keywords:** Atrioesophageal fistula, Catheter ablation, Atrial fibrillation, Meningitis, Septic emboli

## Abstract

Atrioesophageal fistula is a rare, devastating complication of atrial fibrillation ablation, reportedly occurring in 0.015–0.04% of catheter ablations. A 66-year-old African American male with a past medical history of chronic atrial fibrillation status post recent radiofrequency ablation and on chronic anticoagulation with rivaroxaban presented with left upper extremity numbness, tingling, and transient weakness. He was admitted for a cerebrovascular accident workup; a 12-lead electrocardiogram revealed atrial fibrillation and magnetic resonance imaging of the brain was consistent with multifocal embolic infarcts. Hospital course was further complicated by persistent high-grade fevers, gram-positive bacteremia, and worsening mental status requiring mechanical ventilation. Lumbar puncture was consistent with bacterial meningitis. Transthoracic echocardiogram was negative for vegetations. Computed tomography angiography of the chest with intravenous contrast revealed an outpouching off the posterior wall of the left atrium at the level of the inferior pulmonary vein, consistent with an atrioesophageal fistula. We present this case to highlight the clinical features of a rare but potentially fatal complication from a commonly performed procedure requiring prompt recognition and life-saving intervention.

## Highlights

• Atrioesophageal fistula (AEF) is a rare, devastating complication of atrial fibrillation ablation.
• AEF-related complications vary in presentation and should be suspected within three-months of radiofrequency ablation.
• Repeat imaging is reasonable in patients with high clinical suspicion.
• Treatment options include surgical repair and conservative management with esophageal stenting.

## 1 Introduction

Radiofrequency ablation (RFA) for atrial fibrillation (AF) management has emerged as the standard of care for symptomatic persistent and paroxysmal atrial fibrillation refractory to antiarrhythmic therapy. The incidence of atrioesophageal fistula (AEF) after catheter ablation is exceedingly rare, occurring in 0.015%–0.04% of patients [1], while the mortality rate is reportedly 40–100% [2]. While the underlying mechanism remains unclear, it is proposed that thermal injury to the anterior esophageal arteries lying in close proximity to the posterior wall of the left atrium and pulmonary veins leads to ischemia and ulceration of the esophageal mucosa and subsequent fistulization [3]. Patients with AEF formation often have variable presentations leading to diagnostic challenges, delayed surgical intervention, and subsequently increased morbidity and mortality. Neurological complications including grand mal seizures, postprandial transient ischemic attacks, embolic strokes, and meningitis are among the most feared [4]. The exact incidence of meningitis as a complication of AEF remains unknown. We present this case to explore AEF formation and meningitis after RFA, as well as to emphasize the importance of prompt diagnosis and treatment given the high rate of mortality.

## 2 Case report

A 66-year-old African American male with a history of atrial fibrillation, chronically anticoagulated with rivaroxaban, presented to the Emergency Department (ED) with transient left upper extremity numbness, tingling, and weakness 25 days after a percutaneous RFA for atrial fibrillation. Review of systems was largely negative, with the exception of nausea and vomiting 5 days prior to admission. Reported surgical history was limited to atrial fibrillation ablation (6 years prior) and left total hip arthroplasty. Family history was significant for hypertension, hyperlipidemia, coronary artery disease, breast cancer (sister), and colorectal cancer (brother). The patient denied tobacco, alcohol, or illicit drug use. Upon initial evaluation, NIH Stroke Scale Score was 0. Physical examination revealed a fit male appearing younger than his stated age, regular heart rate and rhythm, and a non-focal neurological exam. Cranial nerves II-XII were intact, pupils were equally round and reactive to light and accommodation, speech was normal, and no motor, sensory, or cerebellar testing deficits were appreciated. Labs on admission were significant for leukocytosis of 12,100 cells/mm^3^ and creatinine of 1.74 mg/dL. Initial computed tomography (CT) of the head and magnetic resonance imaging (MRI) of the brain did not show any acute abnormalities suggestive of ischemia or hemorrhage. He was admitted to the general medical floor for a cerebrovascular accident workup. On day two of hospitalization, he developed high-grade fevers up to 103.2 °F [39.6 °C] with remitting-relapsing neurological changes including transient left sided hemiparesis and worsening mental status. Blood cultures were obtained prior to starting empirical treatment with vancomycin, ceftriaxone, ampicillin, and acyclovir for meningitis.

**Fig. 1:**
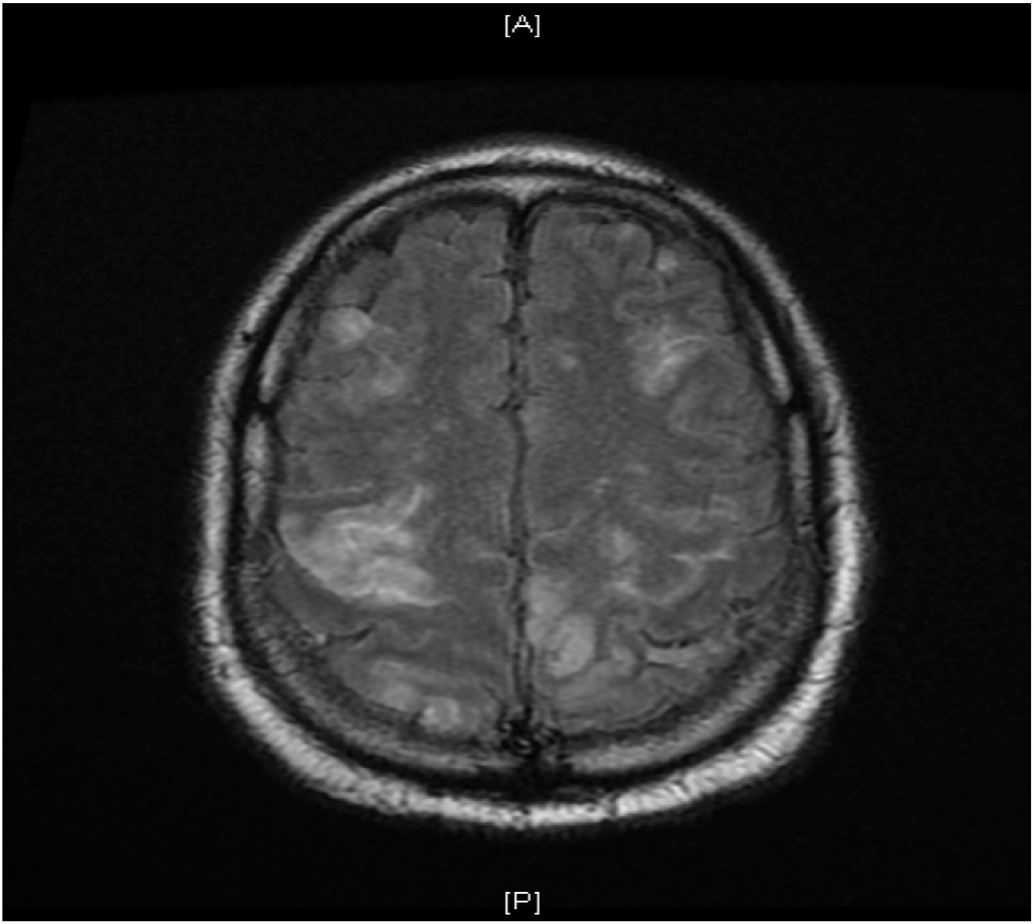
MRI Brain (T2 FLAIR) with multifocal embolic infarcts.

His mental status continued to deteriorate, prompting transfer to the Intensive Care Unit (ICU) for intubation and airway protection. Blood cultures grew gram-positive cocci in chains, which speciated to *Streptococcus pneumoniae*. Transthoracic echocardiogram (TTE) revealed a normal ejection fraction without any other significant abnormalities or vegetations. Repeat CT head showed new questionable areas of infarct. A repeat MRI of the brain revealed multiple embolic infarcts throughout all vascular distributions concerning for an endovascular source (Fig. 1). Lumbar puncture drained cerebrospinal fluid (CSF) with a cloudy appearance, leukocyte count of 1175 cells/mm^3^ with 96% polymorphonuclear leukocytes, total protein 216 mg/dL, and glucose 36 mg/dL, indicating bacterial meningitis. Patient was continued on vancomycin and ceftriaxone.

**Fig. 2:**
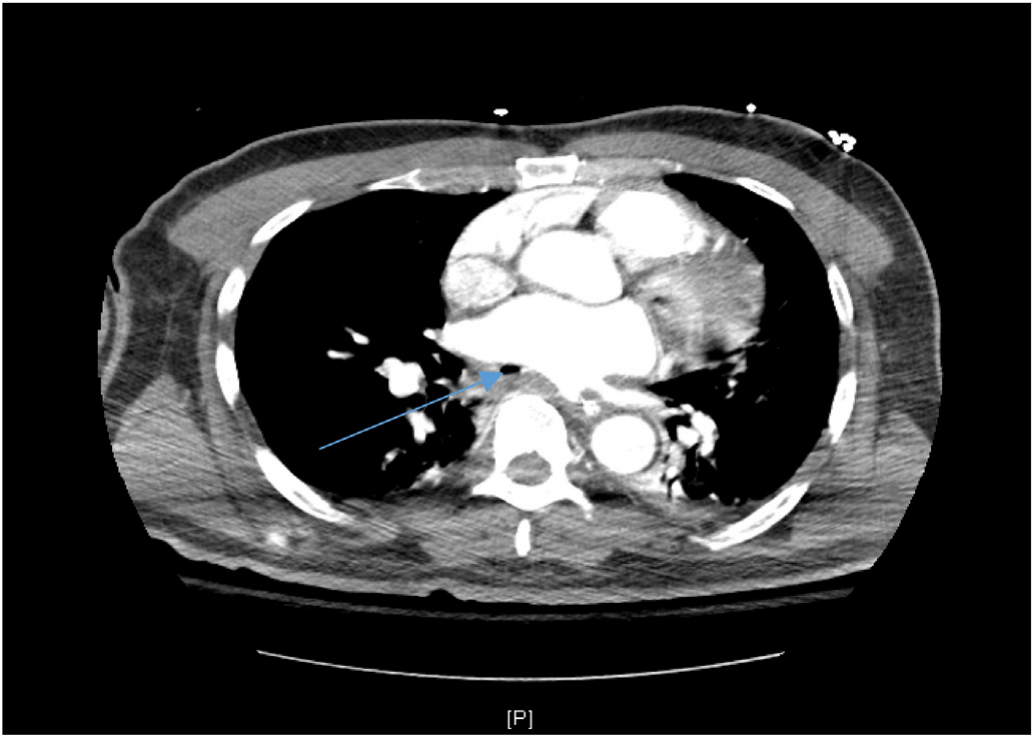
CTA chest with IV contrast demonstrating an outpouching off the posterior wall of the left atrium at the level of the inferior pulmonary vein, consistent with an atrioesophageal fistula (blue arrow). (For interpretation of the references to color in this figure legend, the reader is referred to the Web version of this article.)

Due to persistent AF and concern for an endovascular source of embolic infarcts, he was anticoagulated with a heparin drip. On hospital day 4, he developed upper gastrointestinal (GI) bleeding, prompting discontinuation of heparin. Repeat TTE showed a reduced ejection fraction with global hypokinesis consistent with septic cardiomyopathy. Comprehensive chart review subsequently identified a radiofrequency catheter ablation, performed at an outside facility, approximately 25 days prior to the patient's present admission. Unfortunately, the patient failed to report his recent procedure, and his family was also largely unaware. The combination of bacteremia and multiple cerebrovascular embolic events, in the setting of recent cardiac ablation, raised concerns for AEF. This prompted a stat computed tomography angiography (CTA) of the chest with intravenous (IV) contrast; which ultimately revealed an outpouching off the posterior wall of the left atrium at the level of the inferior pulmonary vein, consistent with an AEF (Fig. 2). The patient was immediately transferred to a tertiary care facility for emergent surgical repair. Prior to surgery the patient remained intubated on minimal ventilator settings responding to painful stimuli only. He maintained hemodynamic stability and was optimized with continued antibiotics and IV fluids. Through a posterolateral thoracotomy the AEF between the mid-esophagus and left atrium was disconnected and the esophageal and left atrial injuries were repaired. Due to persistent hypoxemia when weaning the cardiopulmonary bypass, extracorporeal membrane oxygenation (ECMO) was initiated and discontinued after four days. The patient underwent tracheostomy and jejunostomy-tube placement. He showed minimal neurologic improvement, remaining ventilator dependent with a Glasgow Coma Scale of 7; remarkable for withdrawal to painful stimuli and spontaneous eye-opening, without significant response to verbal stimuli. Left-sided facial droop with left-gaze deviation was also appreciated on final examination. Per his power of attorney, the patient was ultimately discharged to a skilled nursing facility.

## 3 Discussion

During RFA, a catheter is introduced into the left atrium and heat (radiofrequency) is applied to the ectopic myocardial tissues to destroy or scar the tissue contributing to ectopy. This procedure has offered great success while the incidence of procedure-related complications including cardiac tamponade, pericardial effusion, transient ischemic attack, cerebrovascular accident, significant pulmonary vein stenosis and AEF occur in less than 3% of patients [5]. AEF formation is the most feared complication with mortality rates quoted up to 100% [6]. Despite the potentially devastating outcomes associated with AEF, it is rarely reported, poorly understood, and is estimated to occur in less than 0.1% of AF ablation procedures [6].

The anterior esophageal nerve plexus and esophageal arteries pass millimeters from the pulmonary veins and posterior atrial wall making them susceptible to injury [3]. Esophageal temperature monitoring during RFA may lead to early recognition and avoidance of thermal injury [7,8]. In a retrospective study by Knopp et al., an upper endoscopy 1–3 days after left atrial RFA in 425 consecutive patients revealed esophageal erythema in 21%, reflux esophagitis in 12%, and thermal esophageal lesion in 11% [9]. The injury sequelae of arterial ulceration compounded by esophageal dysmotility leads to poor healing, ulcer progression, and fistula formation.

AEF often manifests with non-specific signs and symptoms and a variety of initial presentations including cardioembolic strokes, endocarditis, meningitis, gastrointestinal bleed, and seizures [1,10]. Timing of symptom onset after cardiac ablation is a critical detail to aid in diagnosis. In a global survey of 191,215 AF ablations, responding physicians noted that patients with atrial, pericardial, and esophageal injury, including fistula formation, had symptom onset on postoperative day 19.3 ± 12.6 with a range of 6–59 days [11]. A large systematic review of post-ablative AEF formation similarly described a median time to presentation of approximately 21 days (range: 0–60 days) [10]. In a review of 5 patients who developed esophageal perforation after intraoperative or percutaneous atrial fibrillation ablation, the leading symptom was high fever (n = 3) or severe chest/epigastric pain (n = 2). Leukocytosis was the earliest and most sensitive laboratory marker [12]. In another review of 28 AEF cases, only 5 patients did not experience any neurological complications [4]. Physicians should maintain a high clinical suspicion if patients present with the above mentioned symptoms within 3 months of an AF ablation.

Once an AEF is suspected, radiological evaluation is needed to confirm fistula presence. CT chest with IV contrast should be performed to evaluate for extravasation of contrast from the left atrium into the esophagus [12]. Other suggestive findings on imaging include pneumomediastinum, air in the left atrium or left atrial wall, pleural or pericardial effusions, and esophageal thickening [13]. Repeat imaging should be obtained if a high clinical suspicion remains despite a negative initial chest CT. Han et al. concluded that repeat imaging 4–12 days after an unremarkable initial CT chest was required to establish a definitive diagnosis in 7% of cases [10]. Upper endoscopy is specifically contraindicated, given elevated risk of air embolization, neurological injury, and death [6].

Treatment options for AEF include surgical repair and conservative management via esophageal stenting. Observational studies suggest a mortality benefit of primary surgical repair over endoscopic stenting, although the data is conflicting [6]. Esophageal stenting is particularly useful in cases with left atrial wall friability, which often accompanies AEF formation [3]. Given the rarity of fistulization, a uniform surgical approach to corrective therapy is lacking. Existing case reports describe successful management via transthoracic extrapericardial resection and repair with or without cardiopulmonary bypass. More recently, novel alternatives employing cervical esophageal ligation and decompression have been described [3]. Interposition of a biological barrier between the esophagus and left atrium may reduce the rate of postoperative complications, including mediastinitis, need for percutaneous gastrostomy-tube placement, esophageal stenting, or death [6]. Ultimately, prompt recognition and diagnosis is imperative as emergent, aggressive surgical intervention reduces the morbidity and mortality associated with post-ablation atrioesophageal fistulization.

## 4 Conclusion

Atrioesophageal fistula is a rare but potentially fatal complication occurring up to three months after atrial fibrillation ablation. Neurological complications including meningitis are among the most feared. Prompt recognition and correct utilization of imaging modalities can lead to an early successful diagnosis and implementation of treatment for an often-fatal disease.

### Provenance and peer review

Not commissioned, externally peer reviewed.

## Annals of medicine and surgery

The following information is required for submission. Please note that failure to respond to these questions/statements will mean your submission will be returned. If you have nothing to declare in any of these categories then this should be stated.

Please state any conflicts of interest.

All authors must disclose any financial and personal relationships with other people or organisations that could inappropriately influence (bias) their work. Examples of potential conflicts of interest include employment, consultancies, stock ownership, honoraria, paid expert testimony, patent applications/registrations, and grants or other funding.

The authors declare no conflict of interest.

Please state any sources of funding for your research.

All sources of funding should be declared as an acknowledgement at the end of the text. Authors should declare the role of study sponsors, if any, in the collection, analysis and interpretation of data; in the writing of the manuscript; and in the decision to submit the manuscript for publication. If the study sponsors had no such involvement, the authors should so state.

## Ethical approval

Research studies involving patients require ethical approval. Please state whether approval has been given, name the relevant ethics committee and the state the reference number for their judgement.

This study was approved by Ethics Committee.

## Consent

Studies on patients or volunteers require ethics committee approval and fully informed written consent which should be documented in the paper.

Authors must obtain written and signed consent to publish a case report from the patient (or, where applicable, the patient's guardian or next of kin) prior to submission. We ask Authors to confirm as part of the submission process that such consent has been obtained, and the manuscript must include a statement to this effect in a consent section at the end of the manuscript, as follows: “Written informed consent was obtained from the patient for publication of this case report and accompanying images. A copy of the written consent is available for review by the Editor-in-Chief of this journal on request”.

Patients have a right to privacy. Patients’ and volunteers' names, initials, or hospital numbers should not be used. Images of patients or volunteers should not be used unless the information is essential for scientific purposes and explicit permission has been given as part of the consent. If such consent is made subject to any conditions, the Editor in Chief must be made aware of all such conditions.

Even where consent has been given, identifying details should be omitted if they are not essential. If identifying characteristics are altered to protect anonymity, such as in genetic pedigrees, authors should provide assurance that alterations do not distort scientific meaning and editors should so note.

Exhaustive attempts have been made to contact the patient's family to obtain informed written consent. In the absence of the above-mentioned, this paper has been sufficiently anonymized in order to avoid harm to the patient and his family.

## CRediT authorship contribution statement

**Kayle Warren:** Conceptualization, Writing - original draft, Writing - review & editing. **Pratishtha Singh:** Conceptualization, Writing - original draft, Writing - review & editing, Data curation. **Austen Stoelting:** Data curation, Conceptualization, Writing - review & editing. **Shawn Esperti:** Writing - review & editing, Data curation. **Kunjan Udani:** Writing - review & editing. **Thaddeus Golden:** Writing - review & editing.

## Declaration of competing interest

None.
